# Severe Hyperammonemic Encephalopathy Following Sustained-Release Sodium Valproate Overdose: A Case Report

**DOI:** 10.7759/cureus.107123

**Published:** 2026-04-15

**Authors:** Shotaro Ban, Chiaki Toida, Maiko Yamazaki, Masafumi Yoshida, Takahiro Onuki, Naoto Morimura

**Affiliations:** 1 Emergency Medicine, Teikyo University School of Medicine, Tokyo, JPN; 2 Emergency and Critical Care Medicine, Shinshu University, Nagano, JPN

**Keywords:** hemodialysis, iatrogenic carnitine deficiency, sodium valproate, toxicology, valproate-induced hyperammonemic encephalopathy

## Abstract

Although valproate-induced hyperammonemia is a common adverse effect, it is rarely associated with potentially fatal encephalopathy. A 22-year-old female with a disturbance of consciousness presented to the emergency department after taking an overdose of sustained-release sodium valproate. Initial laboratory testing demonstrated no evidence of hepatic dysfunction, including normal serum transaminases and coagulation parameters, elevated serum ammonia (88 μg/dL), and serum valproate levels (645 μg/dL). The patient was intubated and underwent gastric lavage, followed by multi-dose activated charcoal and lactulose. On day 2, serum levels of ammonia and serum valproate further increased (240 μg/dL and 959 μg/mL, respectively). After intermittent hemodialysis, serum levels of ammonia and serum valproate normalized. However, on day 5, the patient continued to exhibit disturbances of consciousness and was diagnosed with valproate-induced hyperammonemic encephalopathy. The patient was treated with L-carnitine for iatrogenic carnitine deficiency caused by valproic acid. In severe cases, particularly those with marked neurological impairment and high serum valproate concentrations, hemodialysis and L-carnitine administration should be considered.

## Introduction

Valproic acid (VPA) is widely prescribed for the management of epilepsy, migraine, neuropathic pain, and various psychiatric disorders. Although it is generally considered safe, several adverse effects have been reported, among which valproate-induced hyperammonemia (VIH) is relatively common [1]. In most cases, VIH is asymptomatic and does not necessarily correlate with hepatic dysfunction, which may lead to underrecognition in clinical practice. However, in rare instances, it can progress to valproate-induced hyperammonemic encephalopathy (VHE), a serious condition characterized by impaired consciousness, cerebral edema, and potentially life-threatening neurological deterioration [2].

The underlying pathophysiology of VHE is multifactorial and involves mitochondrial dysfunction, impaired β-oxidation, and disruption of the urea cycle. VPA reduces the availability of carnitine, which is essential for mitochondrial metabolism, thereby impairing ammonia detoxification and promoting the accumulation of toxic metabolites [2-4]. These processes may be further amplified in overdose settings. In particular, toxicokinetic changes such as saturation of protein binding increase the free fraction of VPA, which enhances both its toxicity and its dialyzability [5].

Despite increasing awareness of this condition, several challenges remain in its clinical management. Early diagnosis can be difficult, as hyperammonemia may occur without liver dysfunction and often presents with nonspecific neurological symptoms. In addition, although treatments such as hemodialysis and L-carnitine administration have been described, their optimal indications and timing are not fully established, especially in cases involving sustained-release formulations or delayed clinical progression [3,4,6]. Furthermore, the relationship between biochemical improvement and neurological recovery has not been clearly defined.

In this report, we describe a case of severe VHE following an overdose of sustained-release VPA. This case is notable for delayed neurological deterioration despite initial biochemical improvement. The aim of this report is to present the clinical course and discuss the underlying pathophysiology and management strategies, with particular emphasis on toxicokinetics, the role of extracorporeal treatment, and the dissociation between laboratory findings and neurological status.

## Case presentation

A 22-year-old female weighing 72 kg presented to the emergency department four hours after an intentional ingestion of 833.3 mg/kg (120 tablets of 500 mg) sustained-release VPA and 2 mg/kg (144 tablets of 1 mg) lorazepam. She had a history of migraine for which VPA and lorazepam had been prescribed. There was no known history of liver disease, urea cycle disorder, or other metabolic condition predisposing to hyperammonemia.

**Figure 1:**
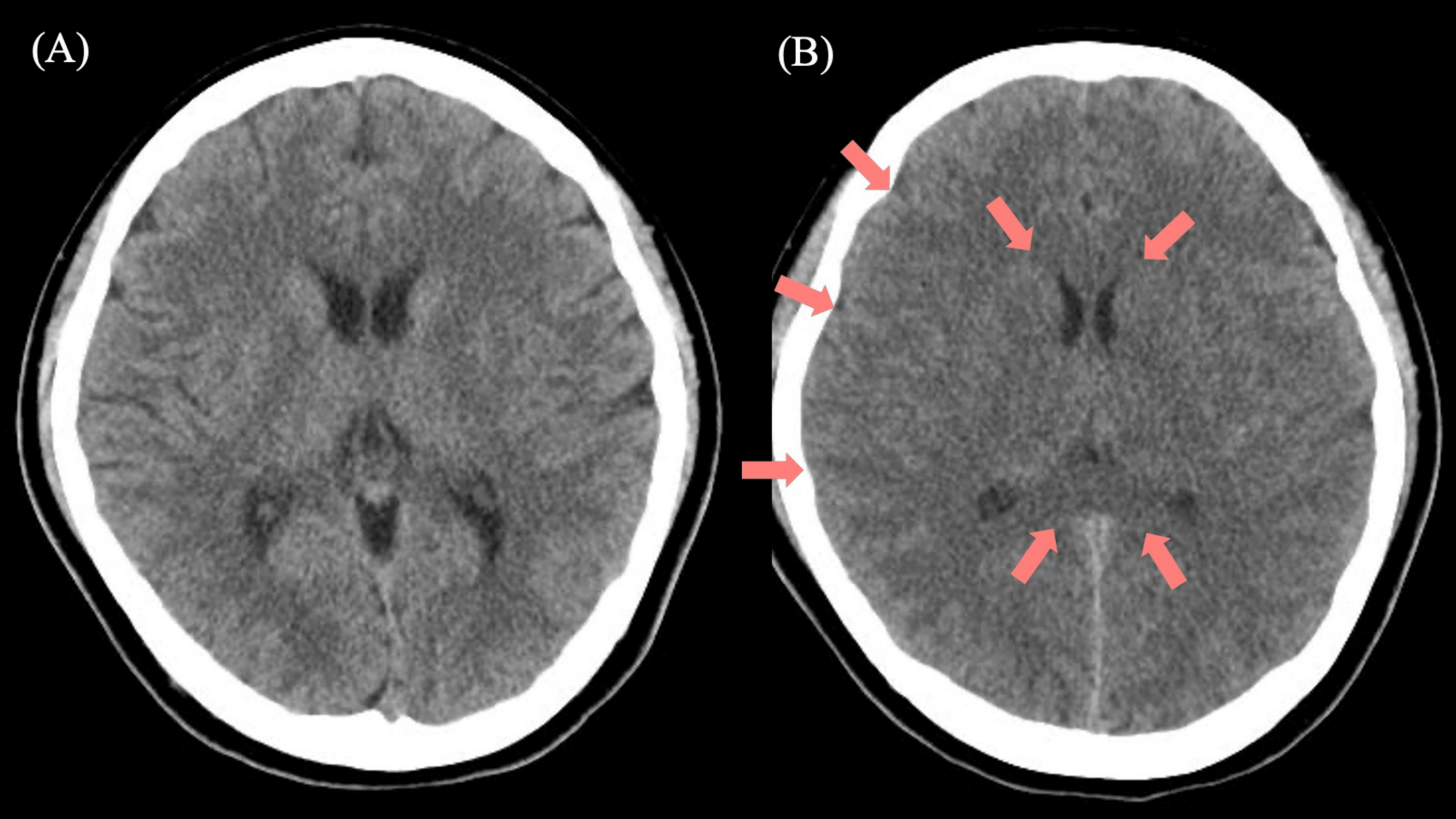
Brain computed tomography (CT) upon admission and on day 5 of admission. (A) Non-contrast axial brain CT upon admission showing no acute abnormalities. (B) Non-contrast axial brain CT on day 5 demonstrating diffuse cerebral edema with effacement of cortical sulci and ventricles, and subtle loss of gray-white matter differentiation (arrows).

Upon arrival, she had markedly depressed consciousness (Glasgow Coma Scale (GCS) score E1V1M5), respiratory depression, and inadequate airway protection, although no seizure activity was observed. Initial vitals noted a respiratory rate of 26 breaths per minute, an oxygen saturation of 77% on a reservoir facemask with 10 L/min of oxygen, a heart rate of 100 beats per minute, blood pressure of 128/86 mmHg, and axillary temperature of 37.4°C. Arterial blood gas analysis revealed a pH of 7.438, PaO_2_ of 108.2 mmHg, PaCO_2_ of 31.5 mmHg, HCO_3_^-^ of 20.8 mmol/l, and lactate of 2.0 mmol/l. Initial laboratory testing demonstrated no evidence of hepatic dysfunction, including normal serum transaminases and coagulation parameters, as well as preserved renal function. The serum ammonia level was 88 μg/dL (reference range, 30-80 μg/dL), and the serum valproate levels were 645 μg/mL (reference range: 50-100 μg/mL). Initial electrocardiography, chest radiography, and brain CT showed no acute abnormal findings (Figure 1A). Due to decreased mental status and lack of airway protection, the patient was intubated and placed on mechanical ventilation. Gastric lavage was performed after airway protection due to massive ingestion of sustained-release VPA and suspected retained gastric contents based on imaging findings, followed by multi-dose activated charcoal (MDAC) and lactulose for valproate toxicity and VIH. The patient was admitted to the intensive care unit for ongoing ventilatory support, close neurological observation, and hemodialysis.

**Figure 2:**
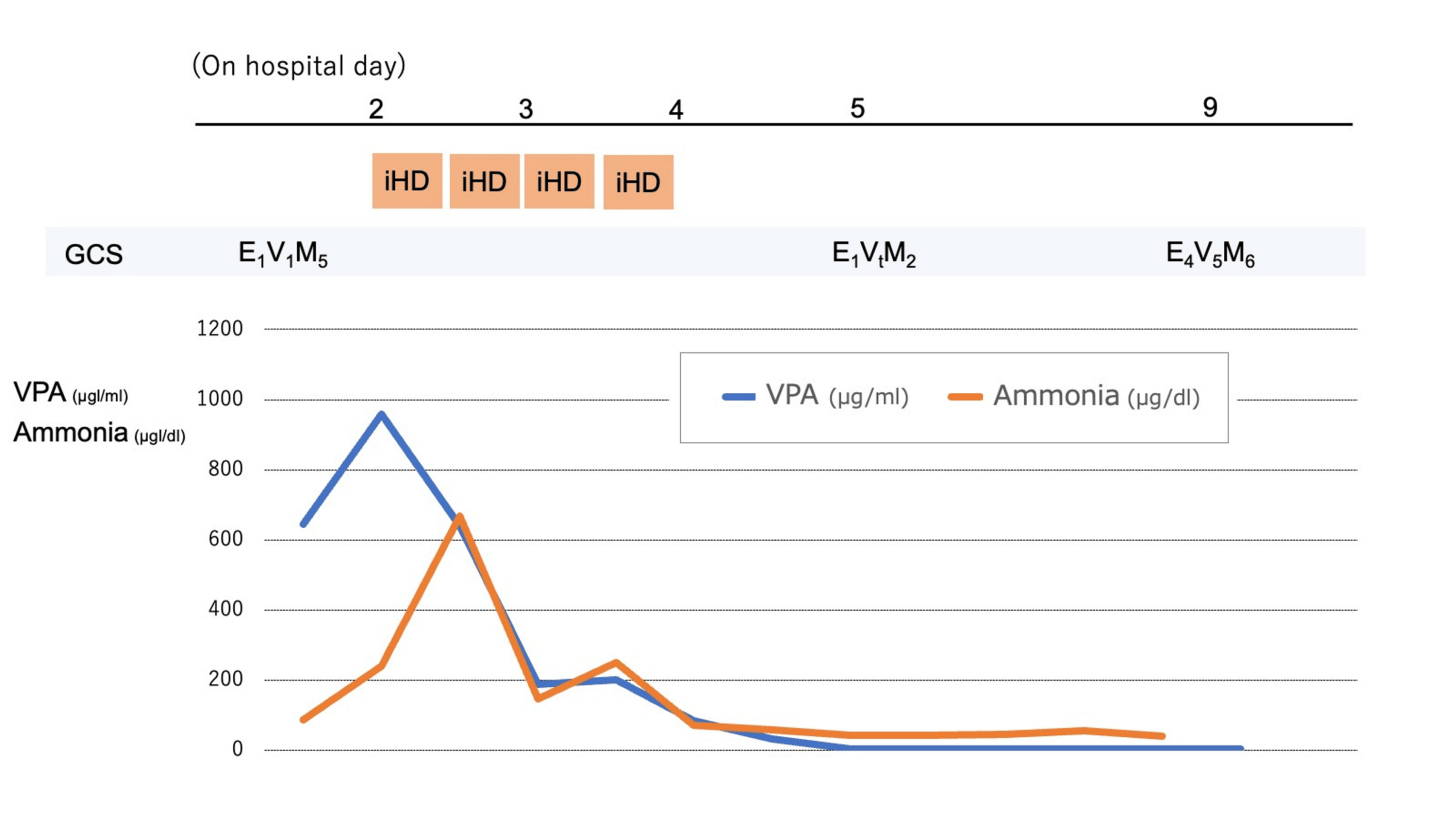
Clinical course of serum valproate levels, ammonia levels, and neurological status. Time course of serum valproate (VPA) and ammonia levels with changes in neurological status (GCS). Intermittent hemodialysis (iHD) was performed, leading to clinical and biochemical improvement.

On day 2, serum levels of ammonia and valproate increased further from 88 to 240 μg/dL and 645 to 959 μg/mL, respectively (Figure 2). Given the rising serum valproate concentration, worsening hyperammonemia, and persistent severe neurological impairment, hemodialysis was initiated in consultation with the neurologist and pharmacist, given clinical features consistent with the Extracorporeal Treatments in Poisoning (EXTRIP) Workgroup recommendation for severe valproate intoxication, including impaired consciousness and hyperammonemia [6]. Intermittent hemodialysis (iHD) was initiated on day 2. The serum levels of ammonia and valproate normalized on day 4 after four cycles of iHD performed every 12 hours. The serum levels of ammonia and VPA at the end of each hemodialysis decreased progressively as follows: ammonia levels (240, 147, 72, and 59 μg/dL) and valproate levels (959, 188, 85, and 33 μg/mL) (Figure 2).

**Figure 3:**
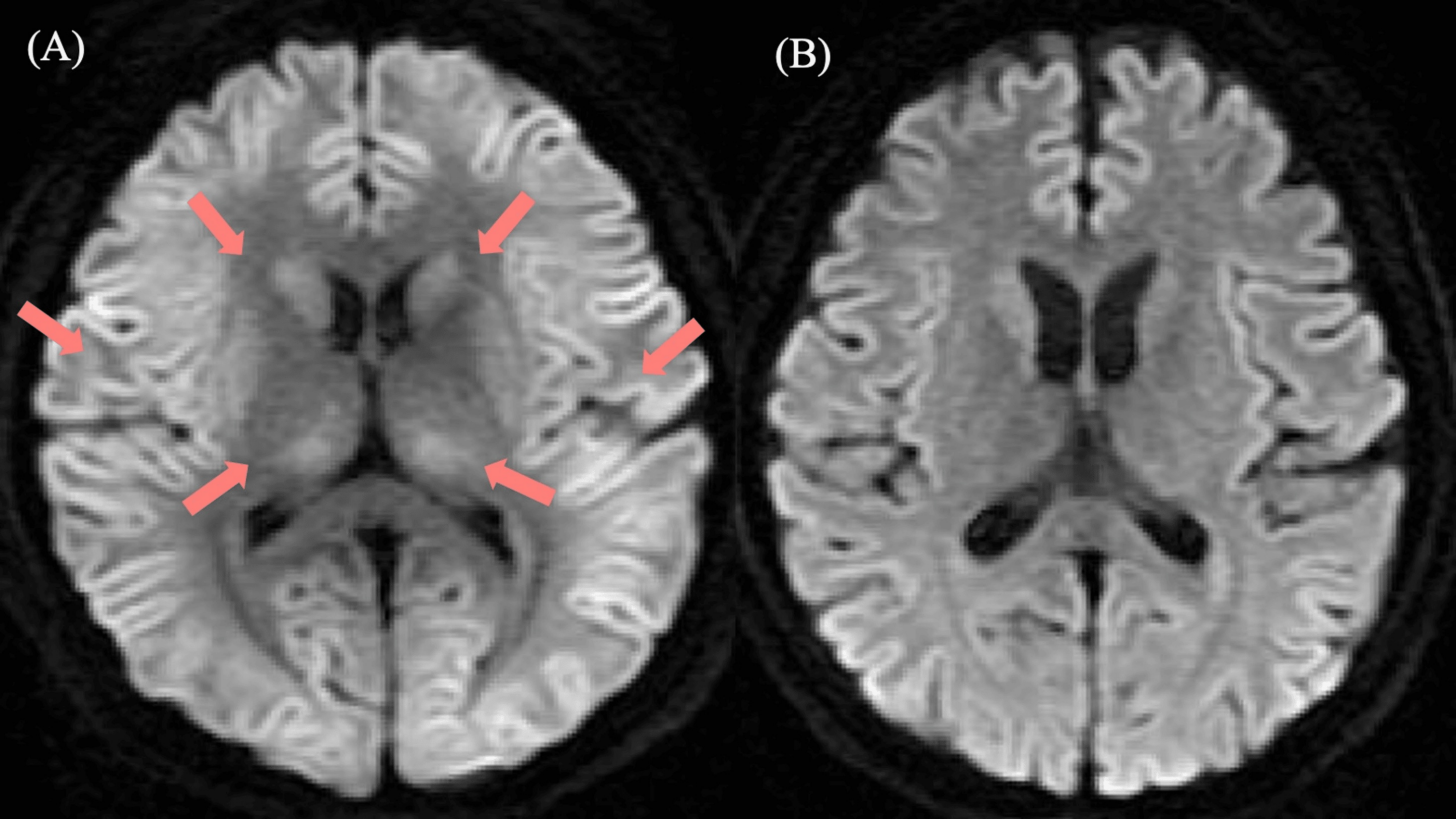
Brain magnetic resonance imaging (MRI) on days 5 and 11 of admission. (A) Axial diffusion-weighted imaging (DWI; b = 1000 s/mm^2^) on day 5 showing diffuse high-intensity signals in the bilateral cerebral cortex and thalami (arrows), consistent with toxic-metabolic encephalopathy. (B) Axial DWI (b = 1000 s/mm^2^) on day 11 demonstrating resolution of the previously observed abnormalities.

**Figure 4:**
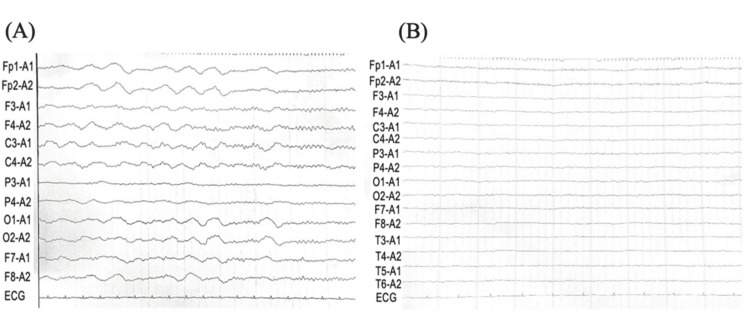
Electroencephalographic (EEG) findings on days 5 and 11 of admission. (A) EEG on day 5 demonstrating high-amplitude 1.5-2 Hz slow waves with intermixed theta activity, consistent with severe encephalopathy. (B) EEG on day 11 showing restoration of background alpha activity (9-10 Hz, 20-30 μV) with intermixed beta activity (15-16 Hz), positive photic driving response, and reduced slow-wave components, corresponding with clinical improvement.

Despite normalization of serum ammonia and valproate levels by day 5, the patient remained deeply comatose (GCS E1V1M2) while still intubated, and the degree of impairment was not fully explained by residual sedation alone, which prompted a repeat brain CT to evaluate for structural or toxic-metabolic cerebral injury. A brain CT scan demonstrated cerebral edema, effacement of cortical sulci and ventricles, and slight loss of gray-white differentiation (Figure 1B). Brain diffusion-weighted magnetic resonance imaging (MRI) revealed diffuse high-intensity areas in the bilateral cerebral cortex, anterior cistern, and thalamus (Figure 3A). EEG on day 5 showed diffuse high-amplitude slow waves with intermixed theta activity (Figure 4A), consistent with severe encephalopathy, whereas EEG on day 11 demonstrated restoration of background alpha activity with reduced slow waves (Figure 4B), corresponding with clinical improvement. The patient was diagnosed with VHE. Additionally, serum carnitine levels were 39.7 to 16.1 μmol/L between days 4 and 5, and L-carnitine was administered on day 5 to treat the iatrogenic carnitine deficiency caused by valproate. L-carnitine was given orally at a dose of 3g per day in three divided doses for 14 days.

After the diagnosis was established, all laboratory tests returned to normal, and the patient began to show signs of improvement. By day 9, she regained full consciousness and successfully weaned from the ventilator. By day 11, abnormalities previously observed in the EEG and brain MRI (Figure 3B) had disappeared. The patient was discharged on day 28 with no apparent neurological deficit.

## Discussion

VIH is primarily mediated through mitochondrial dysfunction, carnitine depletion, and disruption of the urea cycle. Valproate reduces carnitine availability, which is essential for mitochondrial β-oxidation, resulting in impaired ammonia detoxification through inhibition of carbamoyl phosphate synthetase I and accumulation of toxic metabolites [2-4]. In overdose settings, these mechanisms are exacerbated, leading to severe metabolic derangements [2].

The pharmacokinetics of valproate differ significantly in overdose due to saturation of protein binding, resulting in an increased free fraction of the drug. This enhances its dialyzability and contributes to toxicity [5]. Although valproate is highly protein-bound under therapeutic conditions, protein binding becomes saturated at high concentrations, allowing efficient removal by hemodialysis [5,6]. VIH has been reported in 2-80% of patients receiving valproate therapy, although it is asymptomatic in most cases [1]. However, severe cases of VHE, although rare, have been reported and may lead to significant neurological impairment [2]. Several risk factors for VIH have been reported, including high-dose sodium valproate administration, concomitant use of multiple antiepileptic drugs, and underlying metabolic or mitochondrial disorders [7].

Supportive care for acute valproate toxicity includes airway management, stabilization of the general condition, and gastrointestinal decontamination [2]. In this case, gastric lavage and MDAC were performed after airway protection, considering prolonged absorption associated with sustained-release formulations. Although lactulose was administered in this case, its role in VPA-induced hyperammonemia is controversial, and it is not routinely recommended due to potential adverse effects, including volume depletion and increased sodium burden. According to the EXTRIP Workgroup, extracorporeal treatment is recommended in severe valproate poisoning, particularly in patients with markedly elevated serum valproate concentrations, significant neurological impairment, or hyperammonemia [6]. Specifically, EXTRIP recommends extracorporeal treatment in patients with severe clinical features such as coma or cerebral edema, markedly elevated serum valproate levels, and persistent metabolic abnormalities, including hyperammonemia [6]. Ammonia is readily dialyzable due to its low molecular weight and minimal protein binding. Although valproate itself has a relatively low molecular weight and volume of distribution, its high protein binding under normal conditions limits removal; however, this limitation is reduced in overdose settings due to protein-binding saturation [5,6]. Hemodialysis should therefore be considered in patients presenting with severe VHE, particularly when accompanied by high serum valproate levels and neurological impairment [5,6]. L-carnitine is considered a recommended therapy for VPA-induced hyperammonemia, particularly in patients with suspected carnitine deficiency or severe toxicity [3,4].

A notable feature of this case was the persistence of severe encephalopathy despite normalization of serum ammonia and valproate levels after hemodialysis. This dissociation suggests that biochemical improvement alone may not immediately reflect resolution of cerebral toxic-metabolic injury. In our patient, the presence of cerebral edema on CT, diffuse cortical and thalamic abnormalities on MRI, electroencephalographic slowing, and decreased carnitine levels supported ongoing VHE rather than residual sedative effects. Taken together, key diagnostic features of VHE include impaired consciousness, hyperammonemia, elevated serum valproate levels, characteristic neuroimaging findings, electroencephalographic abnormalities, and evidence of carnitine depletion, particularly in the absence of primary hepatic dysfunction [3,4].

Another important consideration is the co-ingestion of lorazepam, which likely contributed to the initial depressed mental status and need for airway protection. However, lorazepam alone would not explain the delayed neurological deterioration, imaging abnormalities, and metabolic findings, which were more consistent with VHE. Furthermore, the temporal relationship between biochemical changes and clinical deterioration is clearly illustrated in Figure 2, which demonstrates the clinical course of serum valproate levels, ammonia levels, and neurological status over time, including the timing of hemodialysis. This visualization supports the clinical decision-making process and highlights the importance of integrating laboratory and clinical findings.

## Conclusions

This case highlights that severe VIHE may occur without liver dysfunction and may persist despite biochemical improvement. Early recognition, close monitoring, and timely initiation of hemodialysis and L-carnitine are important in severe cases. Clinicians should consider VHE in patients with unexplained encephalopathy during valproate toxicity, particularly in the presence of hyperammonemia.
